# MicroRNA-499-5p inhibits transforming growth factor-β1-induced Smad2 signaling pathway and suppresses fibroblast proliferation and collagen synthesis in rat by targeting TGFβ-R1

**DOI:** 10.1007/s11033-023-08755-0

**Published:** 2023-09-07

**Authors:** Qing Zhao, Wentao Yang, Xiangdong Li, Hongtao Yuan, Jianping Guo, Yutang Wang, Zhaoliang Shan

**Affiliations:** 1 Chinese PLA Medical Academy, Beijing, China; 2 Department of Cardiovascular Medicine, The Sixth Medical Center, Chinese PLA General Hospital, Beijing, China; 3 Department of Cardiology, Beijing Jishuitan Hospital, The Fourth Clinical Medical College of Peking University, Beijing, China; 4 Department of Geriatric Cardiology, Chinese PLA General Hospital, Beijing, China

**Keywords:** Atrial fibrosis, Atrial fibroblasts, miR-499-5p, TGFβ-R1

## Abstract

**Background:**

Atrial fibrosis has been recognized as a typical pathological change in atrial fibrillation. Although present evidence suggests that microRNA-499-5p (miR-499-5p) plays an important role in the development of atrial fibrosis, the specific mechanism is not fully understood. Therefore, this study attempted to assess the influence of miR-499-5p on atrial fibroblasts and explore the potential molecular mechanism.

**Methods:**

Atrial fibroblasts from sprague dawley rat were respectively transfected with miR-499-5p mimic, miR-499-5p negative control and miR-499-5p inhibitor, atrial fibroblasts without any treatment were also established. Cell counting kit-8 assay and transwell assay were used to detect the proliferation and migration of atrial fibroblasts in each group. Expressions of miR-499-5p, TGF-β1, smad2, α-SMA, collagen-I and TGFβ-R1 in mRNA and protein level were subsequently detected via quantitative real-time polymerase chain reaction and western blot. Furthermore, the prediction of the binding sites of miR-499-5p and TGFβ-R1 was performed via the bioinformatics online software TargetScan and verified by dual luciferase reporter.

**Results:**

By utilizing miR-499-5p-transfected atrial fibroblasts model, expression of miR-499-5p in the miR-499-5p mimic group was upregulated, while it was downregulated in the miR-499-5p inhibitor group. Upregulated miR-499-5p expression led to a significant decrease in the proliferative and migratory ability of cultured atrial fibroblasts, while downregulated miR-499-5p expression led to a significant increase in the proliferative and migratory ability of cultured atrial fibroblasts. Additionally, upregulated miR-499-5p expression made a significant rise in TGF-β1-induced mRNA and protein expression of TGF-β1, TGFβ-R1, smad2, α-SMA and collagen-I in atrial fibroblasts. Furthermore, results from the dual luciferase reporter conformed that miR-499-5p may repress TGFβ-R1 by binding the 3′UTR of TGFβ-R1 directly.

**Conclusions:**

miR-499-5p is able to inhibit the activation of transforming growth factor β-induced Smad2 signaling and eventually suppressed the proliferation, migration and invasion of atrial fibroblasts and collagen synthesis by targeting TGFβ-R1.

## Introduction

Atrial fibrillation (AF) represents the clinically most common sustained cardiac arrhythmia, and it is a major public health concern due to its rising prevalence, associated thrombotic diseases and myocardial fibrotic diseases, and mortality. Artial fibrosis, which is characterized by the excessive deposition of myocardial interstitial collagen in artial tissue, abnormal distribution as well as the hyperproliferation of cardiac fibroblasts (CFs) has been recognized as a typical pathological change in AF [1–4]. Existing experimental evidence and clinical data indicate that artial fibrosis involves not only in the AF mechanisms but also in AF complications and therapeutic failure [5, 6]. CFs are the most abundant cell type in the heart. They constitute up to 75% of cardiac cells by number but only 10–15% by mass and the activation of them cause process of myocardial fibrosis and AF substrate [7]. It must be noted that the AF substrate associated with extensive cell death and regions of fibrosis may be largely irreversible [8]. Therefore, exploration of the molecular etiology of artial fibrosis remains great importance.

Micro-ribonucleic acids (miRNAs) are endogenous noncoding RNAs containing 18 to 22 nucleotide ribonucleic acid (RNA) sequences that bind to the untranslated regions (most typically the 3′-end) of their target messenger RNAs and regulate gene expression [9]. miRNAs are involved in the regulation of a variety of cardiovascular diseases, including coronary atherosclerosis, AF and heart failure because of their participation in cellular hypertrophy, fibrosis and apoptosis and other physiological or pathological processes [10–13]. miR-499-5p, a family member of miRNA-499, is an evolutionarily and highly enriched microRNA in cardiomyocytes. Prior study has broadcasted that miR-499-5p potentially play a role in the development of atrial fibrillation and acute myocardial infarction [14–16]. However, little progress has been achieved in terms of elucidating the underlying mechanisms of miR-499-5p-mediated atrial fibrosis and its specific fibrosis-related target genes.

Transforming growth factor-β receptor 1 (TGFβ-R1), an irreplaceable downstream molecule of TGF-β1, acts key roles in the TGF-β family. TGF-β1 can participation in proliferation, migration, and collagen secretion of CFs through the TGF-β1/Smads signal transduction pathway. Upon TGF-β1 binding to TGFβ-R2, forming a complex with, they in turn initiate the signal transduction via R-SMADs, which then entered the nucleus to regulate gene expression, thereby resulting cardiac fibrosis [17–19]. In light of these existing discoveries, we hypothesized that miR-499-5p might mediate TGF-β1/Smad2 signal transduction pathway to regulate the function of atrial fibroblasts. Moreover, its target signaling gene was further explored.

## Materials and methods

### Isolation and culture of atrial fibroblasts

SD rats aged 1–3 days (Shanghai SLAC) were killed by cervical dislocation and immersed in 75% alcohol for 10 min. The atria was removed and cut into pieces with sterile eye scissors, and the tissues were centrifuged at 1000 rpm for 5 min in a centrifuge. Afterwards, the tissue samples were transferred into a conical flask, jointly digested with 2 g/L Type II collagenase and 2 g/L trypsin (1:1) for 15 min in 80 rpm in water bath at 37 °C, blowed gently by the straw for 2 min to obtain the single cell, separated cell suspension into 15 mL centrifuge and added high-glucose DMEM (Gibco) supplemented with 10% FBS (Gibco) to terminate the reaction. The digestion was complete when the supernatant was clear. Afterwards, the cell suspension was collected and centrifuged for 5 min at 1000 rpm. After removing the supernatant, the precipitate was incubated in an incubator at 37 °C in an atmosphere of 5% CO_2_. After 60–90 min, the atrial fibroblasts were adhered according to the differential adhesion method. Cells were subcultured for the second or third generation for subsequent experiments. This study was approved by the Animal Ethics Committee of Chinese PLA General Hospital (No. 2021-X17-54). All the procedures in this study were performed in accordance with the institutional guidelines for the care and use of animals. A protocol was prepared before the study without registration.

### Immunofluorescence analysis

Cells are resuspended with complete memum after digested with trypsin. Covered the sterilized 12-well plate with circular coverslips without overlapping. Next, Dripped the cell suspension onto each circular coverslips. 30 min after adding complete culture solution in tissue culture dishes, the atrial fibroblasts were incubated in an incubator (Sanyo) at 37 °C in an atmosphere of 5% CO_2_. Rinsed cells with PBS (3 × 2 min), fixed with 4% polymethylene for 60 min at room temperature. When rinsed well with PBS (3 × 2 min), the coverslips were permeabilized with 0.5% Triton X-100 in PBS for 20 min at room temperature and then treated with 3%  H_2_O_2_ for 15 min. When washes were done, the samples were incubated with alpha smooth muscle (α-SMA, 1:1000, ab7817; Abcam) and vimentin (1:500, ab137321, Abcam) antibodies overnight at 4 °C. Following the primary antibodies incubation, the samples were washed and incubated with with secondary antibodies away from light at room temperature for 2 h. DAPI staining was done and the image was observed using a laser scanning confocal microscope (Leica), with 200× magnifications randomly selected from each sample. The test was done in triplicate.

### Cell grouping and transfection

Atrial fibroblasts at the logarithmic growth phase were removed and assigned into four groups: Control (atrial fibroblasts without any treatment), miR-499-5p mimic (atrial fibroblasts transfected with miR-499-5p mimic consisted of chemically synthesized double-stranded RNAs that simulate the action of endogenous miR-499-5p), miR-499-5p inhibitor (atrial fibroblasts transfected with miR-499-5p inhibitor consisted of single-stranded 2′-*O*-methylated oligonucleotides complementary to miR-499-5p and inhibited endogenous miR-499-5p) and miR-499-5p NC (atrial fibroblasts transfected with miR-499-5p negative control consisted of a scrambled RNA sequence, that has no homology to any known mammalian gene). miR-499-5p NC/mimic/inhibitor were purchased from GenePharma (China). Atrial fibroblasts were seeded per well into 6-well plates and cells transfection was performed using Lipofectamine 2000® (Invitrogen) transfection reagents with 50 nM miR-499-5p NC/mimic/inhibitor, respectively. Cells were collected 12 h after transfection for the cell function assay, 24 and 48 h after transfection for quantitative real-time reverse transcription-polymerase chain reaction (qRT-PCR), and 48 h after transfection for western blotting.

### Cell counting kit-8 (CCK-8) assay

Atrial fibroblasts from each group were inoculated on to 96-well plates (Coring) at a final density of 5000 cells per well in an incubator (Corning)at 37 °C in an atmosphere of 5% CO_2_ after being trypsinized and suspended. Six replicates were set up for each experimental group. Then, the plates were removed at 24, 48 and 72 h for the addition of CCK-8 solution at 20 µL/well (Beyotime), followed by another 2 h of incubation at 37 °C, respectively. Absorbance was determined at a wavelength of 450 nm via a microplate reader (Shanghai Kohua ST-360).

### Transwell migration assay

Atrial fibroblasts at the logarithmic growth phase from each group were diluted to 1 × 10^5^ per mL with serum-free medium. After this step, 150 µL cell suspension was loaded into the upper chamber and 600 µL medium containing 20% FBS was added to the lower chamber, respectively. The cells were fixed with methanol for 30 min, followed by 48 h of incubation at 37 °C. Then the liquid was removed from the upper chamber and the sample was placed in a hole containing 600 µL PBS three times. After crystal violet staining at room temperature for 30 min, cells on the top surface of the membrane were wiped off. The invaded cells were counted in at least five random high-power fields via a light microscope (Olympus) at 200× magnification. The average number of migrated cells was used as a measure of migration capacity. Each experiment was repeated three times.

### Quantitative real-time polymerase chain reaction (qRT-PCR)

Total RNA from atrial fibroblasts was extracted with Trizol Reagent (Invitrogen) according to its operating instructions. Concentration and purity of the samples’ total.

 RNA was determined with NanoDrop 2000 spectrophotometer. RNA was diluted proportionally to make a final concentration 200 ng/µL. Next, RNA was reverse-transcribed to cDNA by stem-loop methods with the PrimeScriptTM RT reagent Kit (TAKARA). The primer sequences of miR-499-5p, TGF-β1, α-SMA, collagen-I, smad2 and TGFβ-R1 were designed by Sangon Biotech (Table 1). An ABI PRISM 7500 real-time PCR appliance and SYBR premix Ex Taq (Tiangen) were used to conduct polymerase chain reactions. U6 and GAPDH were used as the internal references for miRNA and mRNA, respectively. Fold changes in the expression of target genes were calculated using 2^−ΔΔCt^ method.

**Table 1:** Primer sequences

Gene	Primer sequences
β-actin-F	GGGTTACGCGCTCCCTCAT
β-actin-R	TGGCCATCTCTTGCTCGAAG
miR-499-5p-F	GGGGTTAAGACTTGCAGTG
miR-499-5p-R	CAGTGCGTGTCGTGGAGT
U6-F	CGCTTCACGAATTTGCGTGTCAT
U6-R	GCTTCGGCAGCACATATACTAAAAT
TGF-β1-F	CT GCTGACCCCCACTGATAC
TGF-β1-R	AGCCCTGTATTCCGTCTCCT
Collagen I-F	TGGTGAGACGTGGAAACCTG
Collagen I-R	CTTGGGTCCCTCGACTCCTA
α-SMA-F	GGAGCATCCGACCTTGCTAA
α-SMA-R	CCATCTCCAGAGTCCAGCAC
Smad2-F	GTGGTGGAGAACAGAATGGAC
Smad2-R	CAGTCCCCAAATTTCAGAGCA
TGFB-R1-F	CTTCTCATCGTGTTGGTGGC
TGFB-R1-R	GCCTGTCTCGGGGAATTAGG

### Western blot

Atrial fibroblasts were dissolved in cold RIPA lysis buffer (Beyotime) containing phenylmethylsulfonyl uoride (Amresco) and centrifuged at 12,000 rpm for 15 min.

Afterwards, the supernatants were collected and the protein quantification was measured using the bicinchoninic acid protein assay kit (Beyotime). Sodium dodecyl sulfate polyacrylamide gel electrophoresis (SDS-PAGE) was used to separated proteins from each sample. Next, the protein was transferred into nitrocellulose membranes, blocked with 5% nonfat dry milk in TBS for 1 h at room temperature, and incubated in a specific primary antibody at 4 °C overnight. The membranes were washed and incubated with the second antibody for 1 h at room temperature. Finally, the membranes were rinsed with PBS buffer three times and the protein bands were visualized via an enhanced chemiluminescence detection system (ChemiQ4600). The primary antibodies included TGF-β1 (1:1000, ab8126; Abcam), α-SMA (1:1000, ab7817; Abcam), Collagen I (1:1000, ab34710; Abcam), smad2 (1:1000, ab179462; Abcam) and TGFβ-R1 (1:1000, ab3871; Abcam) while β-actin (1:1000, ab8226; Abcam) was set as a control. HRP-labelled goat anti-rabbit IgG (H+L) (1:1000, A0208) and HRP-labelled goat anti-mouse IgG (H+L) antibodies (1:2000, A0216) were purchased from Beyotime. Relative expression levels of proteins were normalized to those of the corresponding β-actin bands as the internal reference.

### Target gene prediction and dual luciferase activity assay

The predicetion of the binding sites of miR-499-5p and TGFβ-R1 was performed by TargetScan 7.2 (http://www.targetscan.org/). To verify if TGFβ-R1 is the target gene of miR**-**499-5p, the fragment of the 3′UTR region of wide-type (Wt) and mutant-type (Mut) TGFβ-R1 were PCR amplified, using the following primers (binding sites are underscored, restriction sites of XhoI and NotI are in italics), respectively: TGFβ-R1 Wt forward, 5′-CCG*CTCGAG*CTGAATTCTAAATCTACCTCAAGGATCTAAGGA*GCGGCCGC*TAAACTAT-3′, and reverse, 5′-TAAGAAT*GCGGCCGC*TCCTTAGATCCTTGAGGTAGTTTAGAATTCAG*CTCGAG*CGG-3′. TGFβ-R1 Mut forward, 5′-CCG*CTCGAG*CTGAATTCTAAATCGACAGCTAGGATCTAAGG*AGCGGCCGC*TAAACTAT-3′, and reverse, 5′-ATAAGAAT*GCGGCCGC*TCCTTAGATCCTAGCTGTCGTTTAGAATTCAG*CTCGAG*CGG-3′. Afterwards, the transcript was cloned into the psiCHECK2 vector (Promega). Subsequently, a total of 2 × 10^5^ 293T cells per well were inoculated and co-transfected with either miR-499-5p mimic or miR-499-5p NC using Lipofectamine 2000® (Invitrogen), according to the manufacturer’s guidelines. After transfection for 48 h, luciferase activity was assayed in triplicate according to the manufacturer’s instructions of a DUAL-GLO® Luciferase Detection System (Promega).

### Statistical analysis

Data analysis was carried out by SPSS 23.0. The data was expressed as the average values ± standard deviation, while the variances between different groups were analyzed by Student’s t-test or one-way ANOVA. *P* < 0.05 indicated a statistically significant differences.Pathological experiment results were analyzed by the Image-Pro Plus 6.0 software.

## Results

### Immunofluorescence detection of atrial fibroblasts

**Fig. 1:**
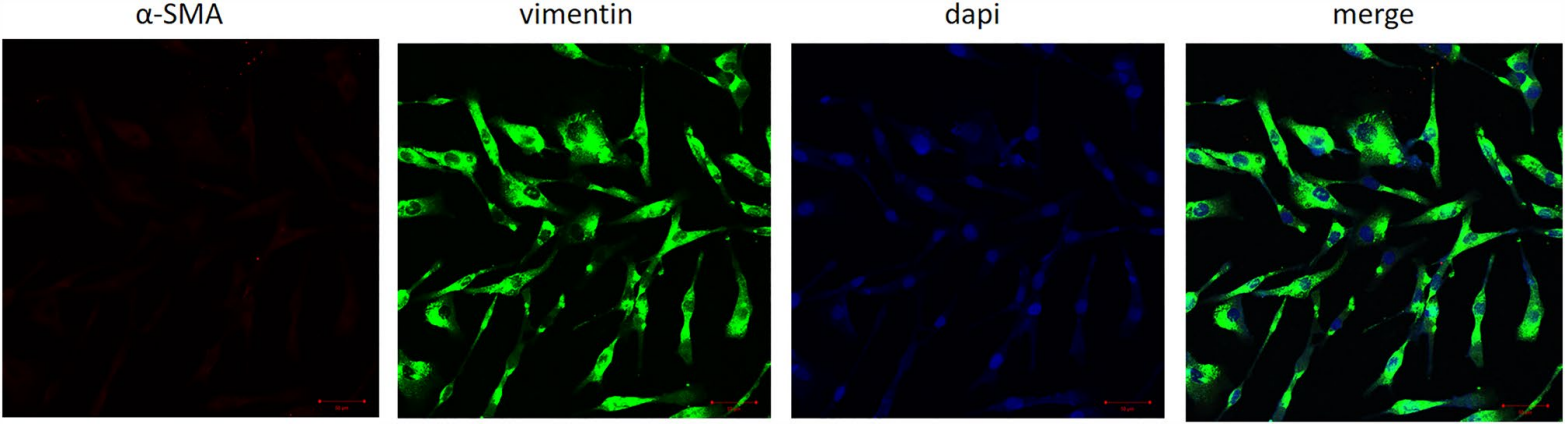
Immunofluorescence detection of atrial fibroblasts by laser scanning confocal microscope. The blue is the nucleus,the green is vimentin and the red is α-SMA

 We adopt α-SMA and vimentin antibodies to identify atrial fibroblasts via a laser scanning confocal microscope. Atrial fibroblasts from rat exhibited spindle or irregular shaped with a large surface area, transparent cytoplasmunder under a microscope. Cells were arranged tightly, and there was a state of overlapping cross growing cells. Result of immunofluorescence detection showed that the expression of vimentin antigen in atrial fibroblasts was positive with the purity of no less than 90% (Fig. 1). This suggests that the cultured atrial fibroblasts had sufficient purity for subsequent experiments.

### miR-499-5p suppressed proliferation and migration of atrial fibroblasts

**Fig. 2:**
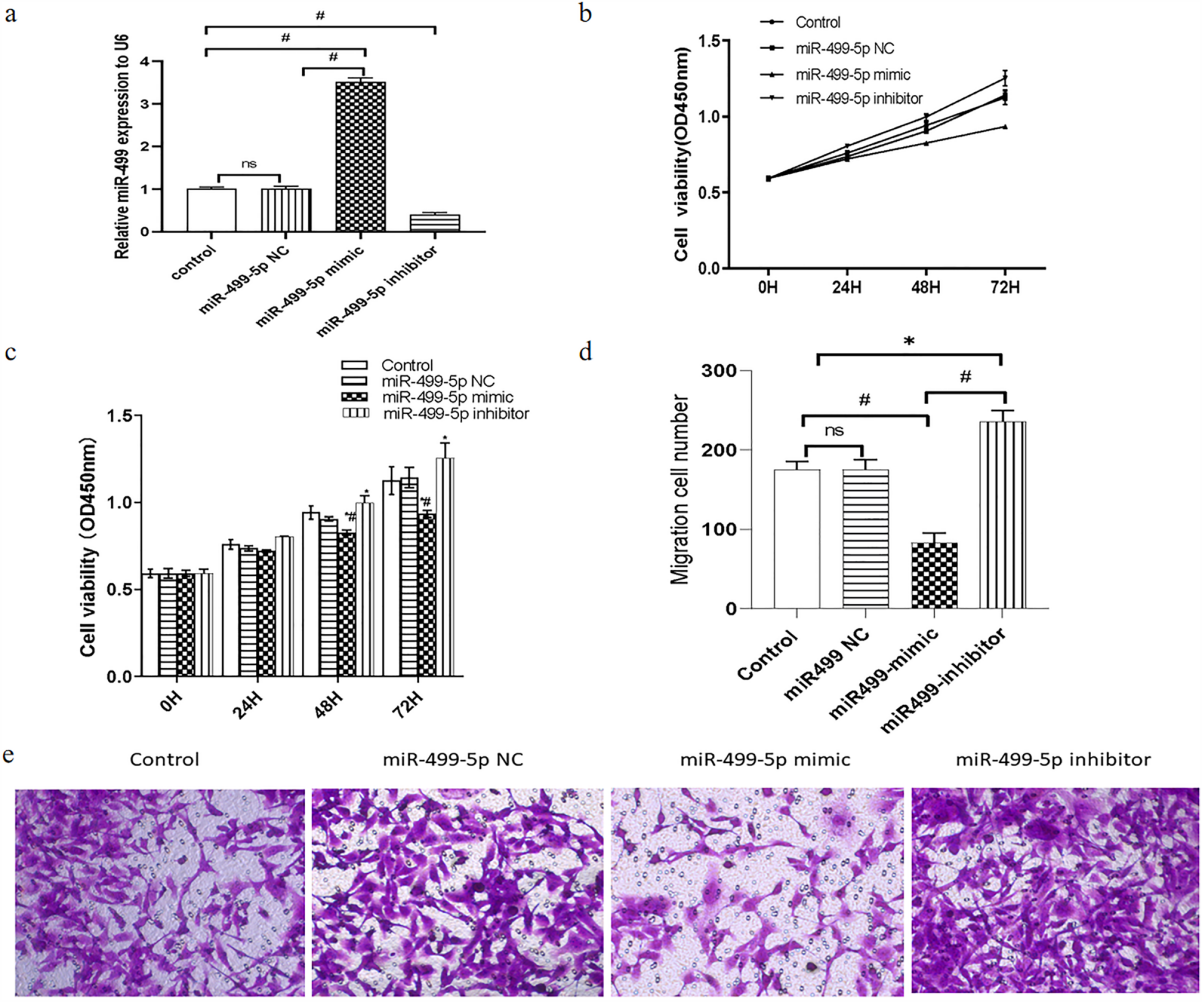
miR-499-5p inhibited proliferation and migration of atrial fibroblasts. a Relative expression of miR-499-5Sp level by qRT-PCR. ^#^P < 0.0001, ns: P > 0.05. **b** Growth curves of atrial fibroblasts detected by CCK assays. **c** The cell proliferation for 0, 24, 48 and 72 h in atrial fibroblasts by CCK assays. *P < 0.05, compared with the control group and the miR**-**499**-**5p NC group, respectively; ^#^P < 0.0001, compared with the miR**-**499**-**5p inhibitor group. **d** The migration cell number by Transwell migration assay. *P < 0.05, ^#^P < 0.0001, ns: P > 0.05. **e** Representative photomicrographs of the migrating cells of the migration cell by Transwell migration assay

 To evaluate the effect of miR-499-5p expression on atrial fibroblasts, miR-499-5p mimic, miR-499-5p NC and miR-499-5p inhibitor were transfected into cultured atrial fibroblasts. Results from qRT-PCR showed that miR-499-5p expression in the miR-499-5p mimic group increased significantly compared with the control and miR-499-5p NC group, while it was reduced significantly in the miR-499-5p inhibitor group compared with the miR-499-5p mimic group (Fig. 2a). Interestingly, the proliferative ability of atrial fibroblasts in the miR-499-5p inhibitor group, the miR-499-5p NC group, the control group, the miR-499-5p mimic group was subsequently weakened, and the absorbance values at OD_450_ nm wave-length at 72 h were: 1.25 ± 0.09, 1.14 ± 0.06, 1.13 ± 0.08, 0.93 ± 0.02, respectively, indicating that upregulated miR-499-5p expression led to a significant decrease in the proliferative ability of atrial fibroblasts (Fig. 2b and c). Meanwhile, Transwell migration assay revealed that the number of migrated cells in the miR-499-5p inhibitor group, the miR-499-5p NC group, the control group, the miR-499-5p mimic group were 245.50 ± 5.24, 179.20 ± 8.82, 181.30 ± 8.36 and 82.67 ± 4.93, respectively, indicating a significant inhibition by miR-499-5p mimic and a significant promotion by miR-499-5p inhibitor on migratory ability of atrial fibroblasts (Fig. 2d and e).

### miR-499-5p inhibited TGF-β1 induced Smad2 signal transduction pathway and collagen secretion in mRNA level

**Fig. 3:**
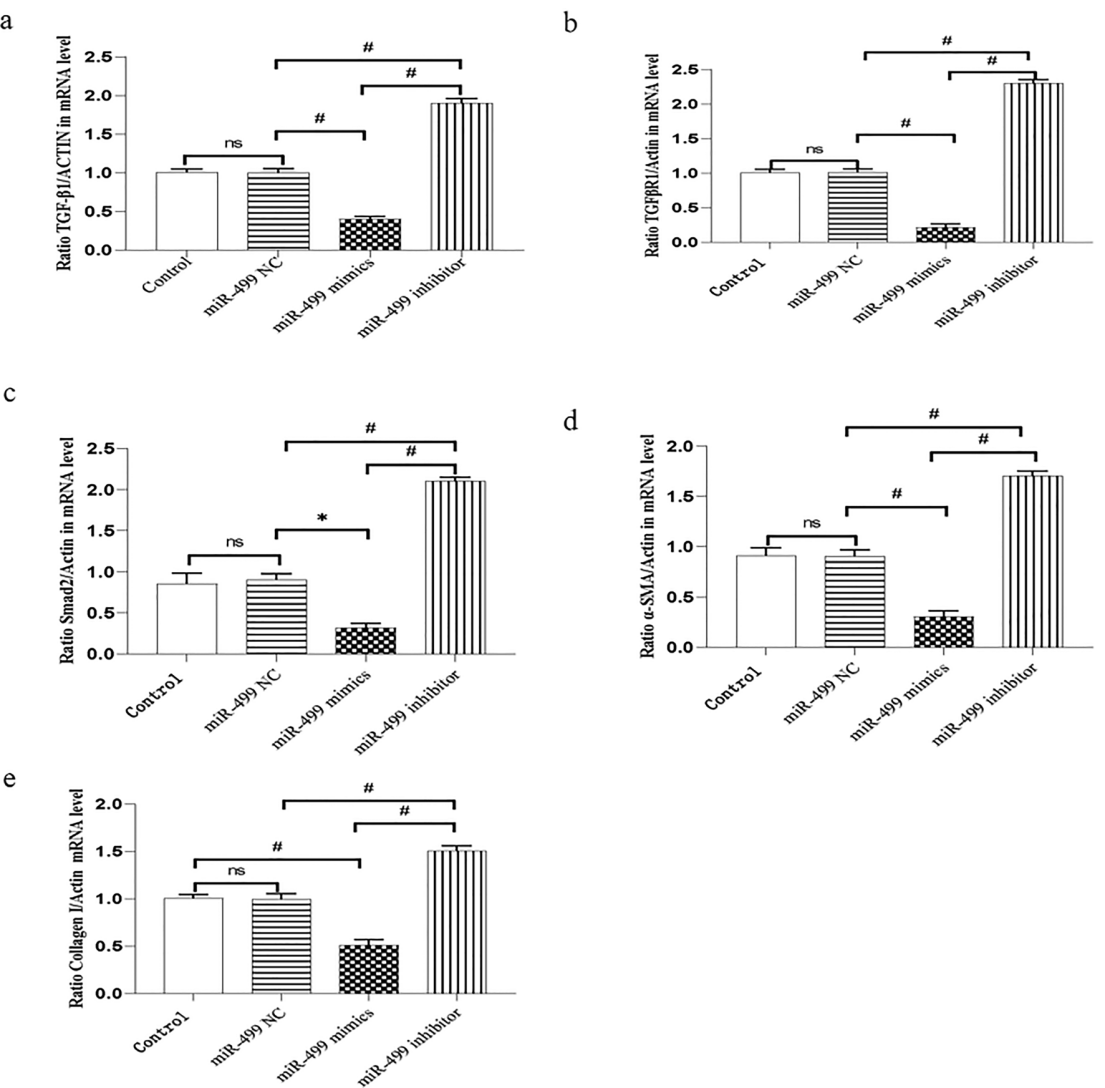
miR-499-5p inhibited the TGF-6/Smads signaling pathway and collagen secretion in mRNA level. **a** Relative expression of TGF-61 level by qRT-PCR. ^#^P < 0.0001, ns: P > 0.05. **b** Relative expression of TGFB-R1 level by qRI-PCR. ^#^P < 0.0001, ns: P > 0.05. **c** Relative expression of Smad2 level by qRI-PCR.*P < 0.001, ^#^P < 0.0001, ns: P > 0.05. **d** Relative expression of a-SMA level by qRI-PCR. ^#^P < 0.0001, ns: P > 0.05. **e** Relative expression of collagen-I level by qRT-PCR. ^#^P < 0.0001, ns: P > 0.05

 To detect the mechanism of the negative regulation of miR-499-5p in atrial fibroblasts, we proceeded to identify the representative TGF-β1/Smad2 signaling pathway and collagen secretion involved in the course. In particular, the results from qPCR showed that the relative expression of TGF-β1, TGFβ-R1, smad2, α-SMA and collagen-I mRNA in the miR-499-5p mimic group were significantly lower compared with the Control and miR**-**499-5p NC group, respectively, while their expression in the miR-499-5p inhibitor group were significantly higher than that of miR-499-5p mimic group (Fig. 3). This demonstrated the participation of TGF-β1/Smad2 signaling pathway in miR-499-5p′s inhibitory effect on fibrotic reply.

### miR-499-5p inhibited TGF-β1 induced Smad2 signal transduction pathway and collagen secretion in protein level

**Fig. 4:**
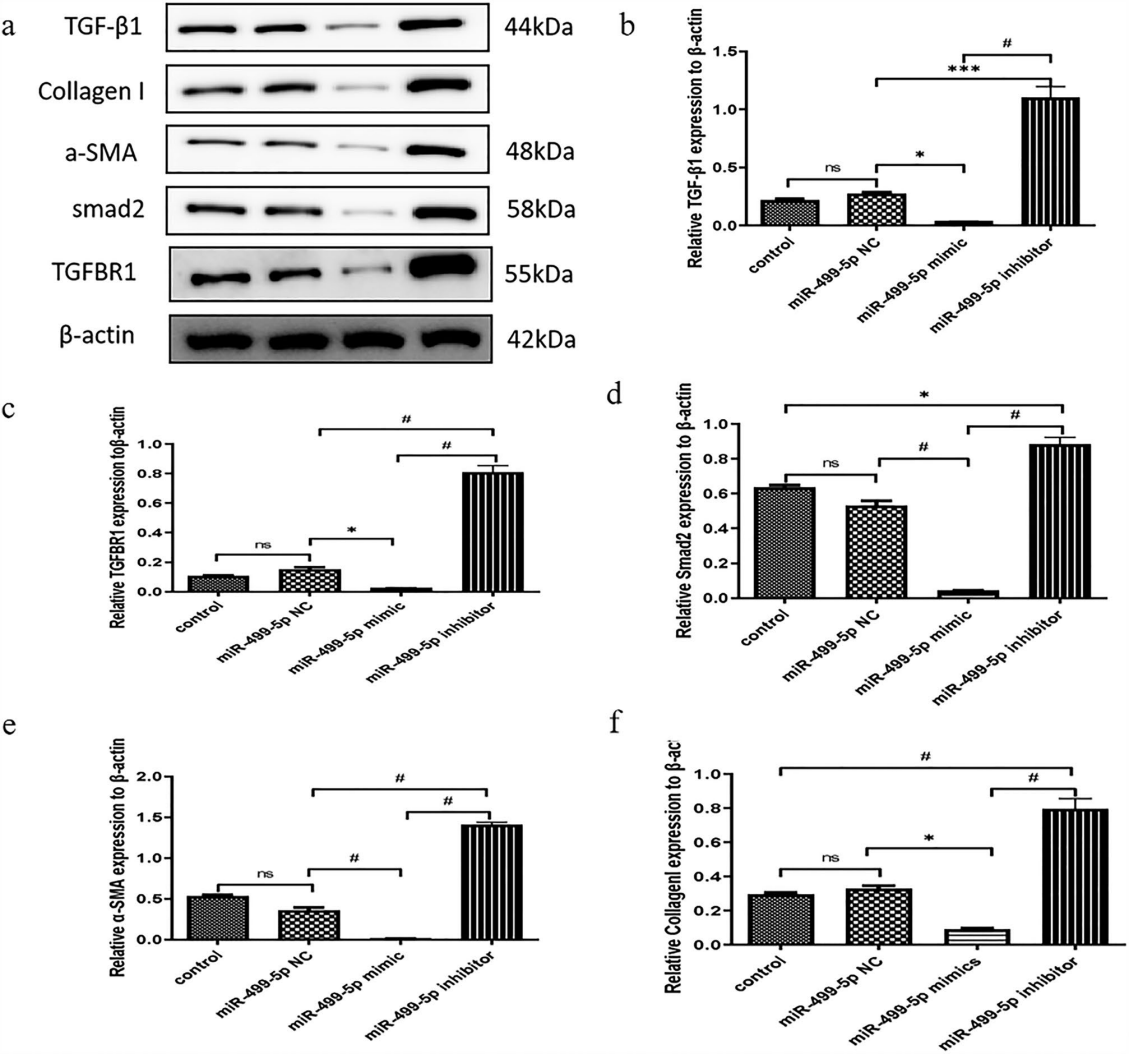
miR-499-5p inhibited the TGF-β1/Smad2 signaling pathway and collagen secretion in protein level. **a** Expression levels of TGF- β1, TGFβ-R1, smad2, α-SMA and collagen-I proteins in atrial fibroblasts. 1, Control group; 2, miR-499-5p NC group; 3, miR-499-5p minic group; 4, miR-499-5p inhibitor group. **b** Relative expression of TGF-β1 level by qRT-PCR. *P < 0.05, ***P < 0.001, ^#^P < 0.0001, ns: P > 0.05. **c** Relative expression of TGFβ-R1 level by qRT-PCR. *P < 0.05, ^#^P < 0.0001, ns:P>0.05. **d** Relative expression of Smad2 level by qRT-PCR. *P < 0.05, ^#^P < 0.0001, ns:P>0.05. **e** Relative expression of α-SMA level by qRT-PCR. *P < 0.05, ^#^P < 0.0001, ns: P>0.05. **f** Relative expression of collagen-I level by qRTPCR. *P < 0.05, ^#^P < 0.0001, ns:P > 0.05

 To detect the mechanism of the negative regulation of miR-499-5p in atrial fibroblasts, we also use Western blot to identify the TGF-β1/Smad2 signaling pathway and collagen secretion involved in the course. The results from Western blot showed that the expression of TGF-β1, TGFβ-R1, smad2, α-SMA and collagen-I protein in the miR-499-5p mimic group were significantly lower compared with the Control and miR-499-5p NC group, respectively, while their expression in the miR-499-5p inhibitor group were significantly higher than that of miR-499-5p mimic group (Fig. 4), which suggested the involvement of the TGF-β1/Smad2 signaling pathway in miR-499-5p′s anti-fibrotic regulatory effect effect on atrial fibroblasts.

### miR-499-5p regulated the expression of TGF-β1/Smad2 signal transduction pathway by targeting TGFβ-R1

**Fig. 5:**
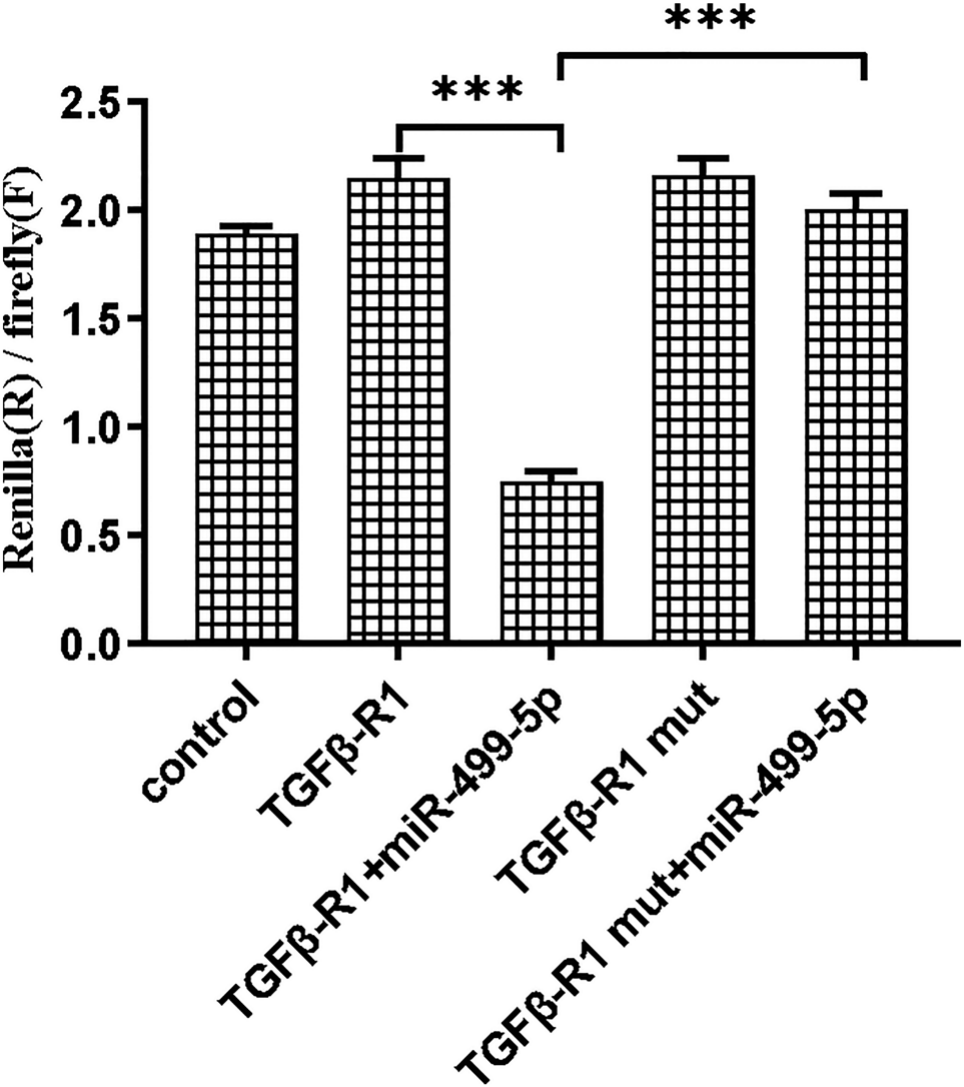
After 293T cells was co-transfection, a dual luciferase report assay was used to detect the luciferase activity. The luciferase activity was significantly decreased in TGFB-R1 Wt+miR-499-5p minic group compared with that in TGFB-R1 Wt and TGFB-R1 Mut+miR-499-Sp minic group. ***P < 0.001

 Based on the above results, we further elucicated the underlying molecular mechanism that miR-499-5p alleviated atrial fibrosis. The publicly-available online software TargetScan 7.2 (http://www.targetscan.org/) was performed for the prediction of its potential target genes. The suggested miRNA binding site in the 3′UTR region of TGFβ-R1Wt type as well as TGFβ-R1 Mut type gene is described in Materials and Methods. The TGFβ-R1 3′UTR was cloned into psiCHECK-2 prior to dual luciferase assay. As indicated in Fig. 5, the Renilla luciferase/Firefly luciferase ratio in HEK 293T cells was significantly decreased (with a reduction of more than 55%) in TGFβ-R1 Wt+miR-499-5p mimics group compared with that in TGFβ-R1 Wt and TGFβ-R1 Mut+miR-499-5p mimics group, respectively. This outcome suggested that miR-499-5p may directly target the 3′UTR of TGFβ-R1.

## Discussion

Up to now, the understanding of AF focus on arrhythmia substrates. Atrial fibrosis, the fundamental feature of atrial structural remodeling, constitutes the arrhythmia substrates in patients suffering from AF, which is characterized by the replacement of dead cardiomyocytes with fibroblasts and subsequent abnormal synthesis and irregular deposition of extracellular matrix (ECM) proteins [1, 3]. Because of the primary contribution of CFs to the synthesis and secretion of ECM, the differences in distribution, aberrant cell migration, proliferation between atrial fibroblasts and ventricular fibroblast [20], and the central role of the migration and proliferation of atrial fibroblasts in AF-promoting structure remodeling, here, we isolated the atrial fibroblasts from the atrial tissue of rats. We observed changes in the abilities of atrial fibroblast migration and proliferation, along with miR-499-5p and its target gene.

First, we observed the influence of miR-499-5p on atrial fibroblasts, and found that the proliferative and migratory ability of cultured atrial fibroblasts was suppressed by miR-499-5p minic, while it was enhanced by miR-499-5p inhibitor. Subsequently, to explore the potential mechanism through which the above phenomenon occurs, we conducted further experiments and found that the miR-499-5p presented a protective ability to inhibit TGF-β1 induced atrial fibroblasts respond, along with the collagen secretion in atrial fibroblasts. Furthermore, results from the TGFβ-R1 3′UTR-luciferase reporter vector verified that miR-499-5p binds to the 3′UTR of TGFβ-R1 mRNA. To the best of our knowledge, our study is the first to reveal that miR-499-5p can inhibits TGF-β1/Smad2 Signaling pathway and suppresses proliferation and migration of atrial fibroblasts of atrial fibroblasts in rat by targeting TGFβ-R1. Taken together, these observations can provide convincing evidence for further research to elucidate the potential anti-fibrotic role of miR-499-5p for the structure remodeling in AF.

More specifically, it is established that the chief components of ECM are pro-proteins, the majority of which is collagen-I [21]. TGF-β1 is involved in cell proliferation and migration as well as the synthesis of ECM and secretion of α-SMA [22]. Aparting from being as an expression protein of fibroblasts, α-SMA also participates in the reconstruction of ECM and influences the occurrence of atrial fibrosis directly [23]. To investigate the effects in our work, we detected the expression levels of collagen-I and α-SMA in atrial fibroblasts with respective treatments.

Recent evidence from a number of in vivo and vitro studies has elucidated that miR-449 participate in the regulation of myocardial fibrosis and apoptosis [10, 13, 15, 16, 24–26]. Ling et al. discovered that atrial miR-499 was significantly upregulated in the atrial tissues from patients with permanent AF, contributing to the electrical remodeling in AF [15, 24]. miR-499-5p, a family member of miR-499, was nearly specifically expressed in cardiac cell. Clinical trails have shown that circulating miR-499-5p was substantially elevated in acute myocardial infarction (AMI) patients [27, 28]. Microarray analysis by Dong et al. found down-regulated miR-499-5p in the infarcted area of rat heart [29]. MiR-449-5p was first verified to be remarkably decreased in the infarcted myocardial tissues than noninfarcted myocardial tissues in established Rat AMI models by Li et al. They further detected a remarkably higher miR-499-5p expression level in cultured neonatal rat cardiomyocytes induced by hypoxia. Additionally, the upregulated miR-499-5p resulted in an obviously decreased myocardial infarct sizes in the rat models due to the reduction of the cardiomyocytes apoptosis [30]. However, the existed works mostly tend to demonstrate a protective effect of miR-499-5p on myocardial infarction during ischemia/reperfusion injury. In the current study, we primarily oberserved that miR-499-5p suppressed the proliferative and migratory ability of cultured atrial fibroblasts. Additionally, upregulation of miR-499-5p resulted in significantly decreased expression of TGF-β1, α-SMA and collagen-I, while downregulation of miR-499-5p resulted in significantly increased expression of TGF-β1, α-SMA and collagen-I, suggesting that promotting miR-499-5p can reduce collagen levels to alleviate atrial fibrosis in some way. Our findings therefore provided novel information on the role of the cellular functions of miR-499-5p in regulation of myocardial fibrosis.

The canonical TGF-β/Smad2/3 signalling pathways plays a key role in the formation of cardiac fibrosis [17, 31]. As reported by Wang et al., miR-27b blocked the Smad2/3 pathway to inhibit atrial fibrosis [32]. Han et al. [18] reported that up-regulating miR-29b could block the TGF-β1/Smad-2/3 signaling pathway to mitigate atrial fibrosis in AF rats Therefore, to better explore the mechanism of miR-499-5p on inhibiting atrial fibrosis in our work, the TGF-βl/Smad2 pathway was measured in the present study. Atrial fibroblasts were isolated and treated with miR-499-5p mimics or inhibitors. Consequently, overexpression of miR-499-5p could downregulate TGF-β1, along with TGFβ-R1 and Smad2, whereas inhibiting miR-499-5p brought out the opposite trend, which suggested that miR-499-5p may inhibit the TGF-βl/Smads2 signaling pathway to alleviate atrial fibrosis. As noted, when the TGF-β ligand binds to TGFβ-R2, and phosphorylates TGFβ-R1, the intracellular Smad2/3 pathway is activated. The phosphorylated Smad2 and Smad3 oligomerize with Smad4 and translocate to the nucleus to regulate expression of TGF-β target genes [33].

As miRNAs modulate their biological functions through modulating several target genes, definitive identification of miRNA target genes is essential. After searching literature, we found that KCNN3, CACNB2, PDCD4, PACS2 and SOX6 were verified to be target genes of miR-499-5p in cardiomyocytes [15, 24, 30, 34, 35]. However, no studies have verified its target genes in atrial fibroblasts. In the present study, we predicted the potential target genes of miR-499-5p by bioinformatic analysis and observed a complementary binding site between miR-499-5p and TGFβ-R1 3′UTR. We then analyzed its role in atrial fibrosis by luciferase reporter assays and found that TGFβ-R1 might be a miR-499-5p target involved in fibrosis. TGFβ-R1, a receptor of TGF-β, is essential to fibrosis in the cardiovascular system and other organs [36]. TGFβ-R1 has been validated as a direct target of miR-130a, and it has been reported that miR-130a exerted its antifibrotic properties by directly targeting TGFβ-R1 to regulate the activity of TGF-β/Smad signaling [37].We carried out the dual-luciferase report assay, confirming that TGFβR-1 was indeed a direct target gene of miR-499-5p because ectopic overexpression of miR-499-5p decreased the luciferase activities of HEK 293T cells transfected with the wild type TGFβ-R1 3′UTR reporter vector rather than the mutant reporter vector.

## Conclusion

The current study revealed the anti-fibrotic role of miR-499-5p in atrial fibroblasts, and gave a demonstration of a link between miR-499-5p, TGF-β1/Smad2 signal transduction pathway and cardiac fibrosis. We found that overexpression of miR-499-5p could reduce the proliferative and migratory ability of atrial fibroblasts in rat, inhibits transforming growth factor-β1-induced Smad2 signaling pathway, along with collagen formation through targeting TGFβ-R1. Modulation of miR-499-5p/TGFβ-R1 levels might present a potential preventive approach for atrial fibosis.

## Limitations

Some limitations of the present work need to be acknowledged. We did not investigate full-scale TGF-β1-involved signaling transduction pathways. Therefore, we evaluated a partial effect of miR-499-5p on inhibiting the TGF-β1/Smad2 signaling pathway. Furthermore, we performed the experiments in vitro, therefore, further studies in animal models and clinical AF individuals are needed to confirm our exploration at a later stage.

## Data Availability

The data underlying this article will be shared on reasonable request to the corresponding author.
