# Correction: MicroRNA‑499‑5p inhibits transforming growth factor‑β1‑induced Smad2 signaling pathway and suppresses fibroblast proliferation and collagen synthesis in rat by targeting TGFβ‑R1

**DOI:** 10.1007/s11033-024-09226-w

**Published:** 2024-04-20

**Authors:** Qing Zhao, Wentao Yang, Xiangdong Li, Hongtao Yuan, Jianping Guo, Yutang Wang, Zhaoliang Shan

**Affiliations:** 1 Chinese PLA Medical Academy, Beijing, China; 2 Department of Cardiovascular Medicine, The Sixth Medical Center, Chinese PLA General Hospital, Beijing, China; 3 Department of Cardiology, Beijing Jishuitan Hospital, The Fourth Clinical Medical College of Peking University, Beijing, China; 4 Department of Geriatric Cardiology, Chinese PLA General Hospital, Beijing, China

**Correction: Molecular Biology Reports (2023) 50:9757–9767** 10.1007/s11033-023-08755-0

In the published article, the word ‘Artial’ is corrected as ‘Atrial’ in the abstract section.

In the section ‘Target gene prediction and dual luciferase activity assay’, the sentence ‘To verify if TGFβ-R1 is the target gene of miR-499-5p, the fragment of the 3′UTR region of wide-type (Wt) and mutant-type (Mut) TGFβ-R1 were PCR amplified, using the following primers (binding sites are underscored, restriction sites of SacI and Hind III are in italics), respectively: TGFβ-R1 Wt forward, 5′-*CGAGCTCG*CTGAATTCTAAATCTACCTCAAGGATCTAA*GGACAA GCTTG*-3′, and reverse, 5′-*CGAGCTCG*TCCTTAGATCCTTGAGGTAGTTTAGAATT*CAGCGAGCTCG*-3′. TGFβ-R1 Mut forward, 5′-*CGAGCTCG*CTGAATTCTAAATCGACAGCTAGGATCTAAGGA*CAAGCTTG*-3′, and reverse, 5′-*CAAGCTT*GTCCTTAGATCCTAGCTGTCGTTTAGAATTCAG*CGAGCTCG*-3′ is corrected as ‘To verify if TGFβ-R1 is the target gene of miR-499-5p, the fragment of the 3′UTR region of wide-type (Wt) and mutant-type (Mut) TGFβ-R1 were PCR amplified, using the following primers (binding sites are underscored, restriction sites of XhoI and NotI are in italics), respectively: TGFβ-R1 Wt forward, 5′-CCG*CTCGAGC*TGAATTCTAAATCTACCTCAAGGATCTAAGGA*GCGGCCGC*TAAACTAT-3′, and reverse, 5′-TAAGAAT*GCGGCCGC*TCCTTAGATCCTTGAGGTAGTTTAGAATTCAGCTCGAGCGG-3′. TGFβ-R1 Mut forward, 5′-CCG*CTCGAGC*TGAATTCTAAATCGACAGCTAGGATCTAAGGA*GCGGCCGC*TAAACTAT-3′, and reverse, 5′-ATAAGAAT*GCGGCCGCTCC*TTAGATCCTAGCTGTCGTTTAGAATTCAG*CTCGAG*CGG-3′.

As the 18th reference ‘Li PF, He RH, Shi SB, Li R, Wang QT, Rao GT, Yang B (2019) Modulation of miR-10a-mediated TGF-β1/Smads signaling affects atrial fibrillation-induced cardiac fibrosis and cardiac fibroblast proliferation. Biosci Rep 39(2):BSR 20181931’ was retracted. Hence, it has been replaced with ‘Han et al. [18] reported that up-regulating miR-29b could block the TGF-β1/Smad-2/3 signaling pathway to mitigate atrial fibrosis in AF rats’.

In the discussion section, the sentence ‘Li et al. [18] reported that down-regulating miR-10a could block the TGF-β1/Smads signaling pathway to decrease atrial structural remodeling’ is corrected as ‘Han et al. [18] reported that up-regulating miR-29b could block the TGF-β1/Smad-2/3 signaling pathway to mitigate atrial fibrosis in AF rats’.

The Fig. 3 is updated with the identification of a group.

**Fig. 3:**
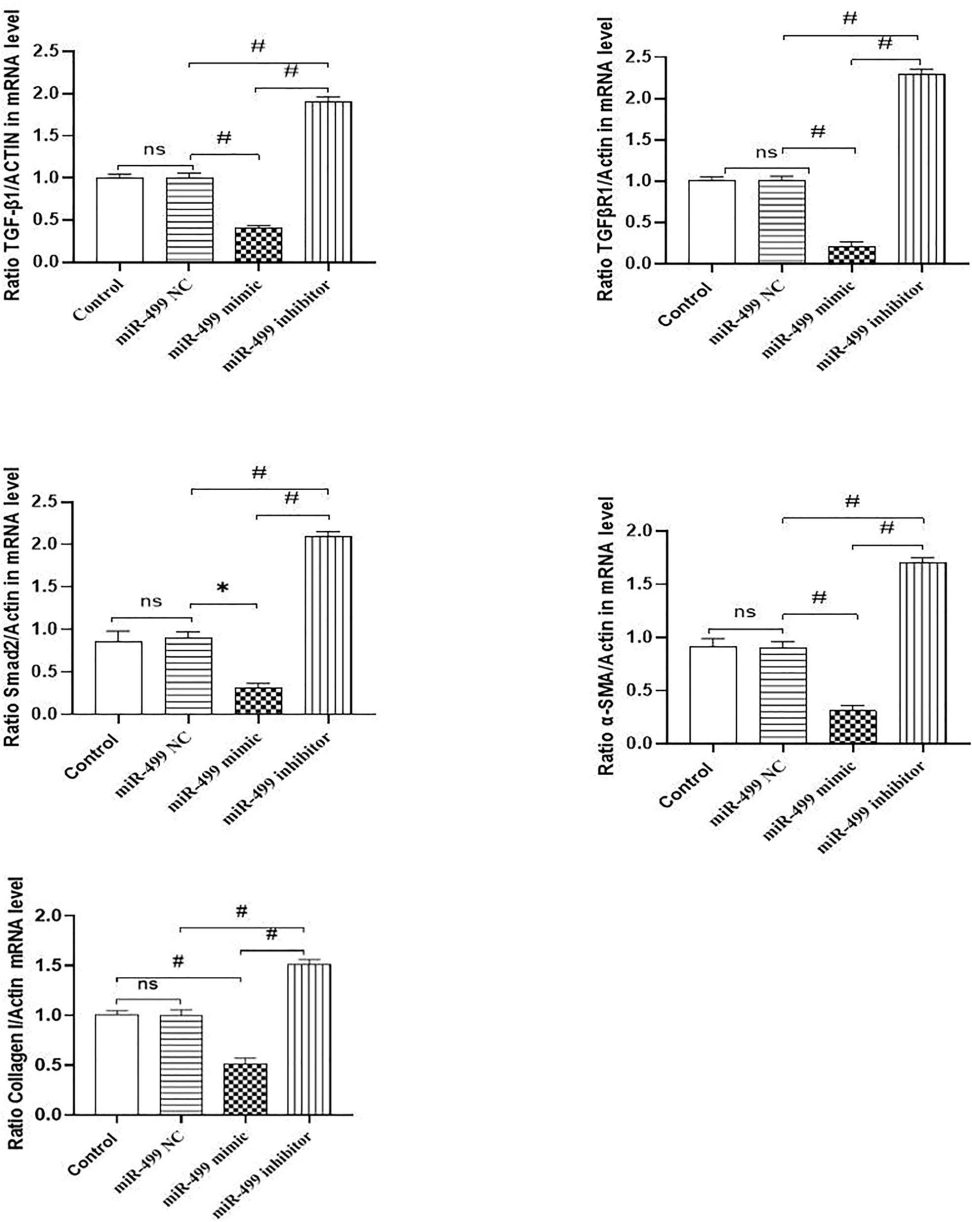
miR-499-5p inhibited the TGF-6/Smads signaling pathway and collagen secretion in mRNA level. **a** Relative expression of TGF-61 level by qRT-PCR. ^#^P < 0.0001, ns: P > 0.05. **b** Relative expression of TGFB-R1 level by qRI-PCR. ^#^P < 0.0001, ns: P > 0.05. **c** Relative expression of Smad2 level by qRI-PCR.*P < 0.001, ^#^P < 0.0001, ns: P > 0.05. **d** Relative expression of a-SMA level by qRI-PCR. ^#^P < 0.0001, ns: P > 0.05. **e** Relative expression of collagen-I level by qRT-PCR. ^#^P < 0.0001, ns: P > 0.05

The Fig. 4 is updated with the missing molecular weight of collagen.

**Fig. 4:**
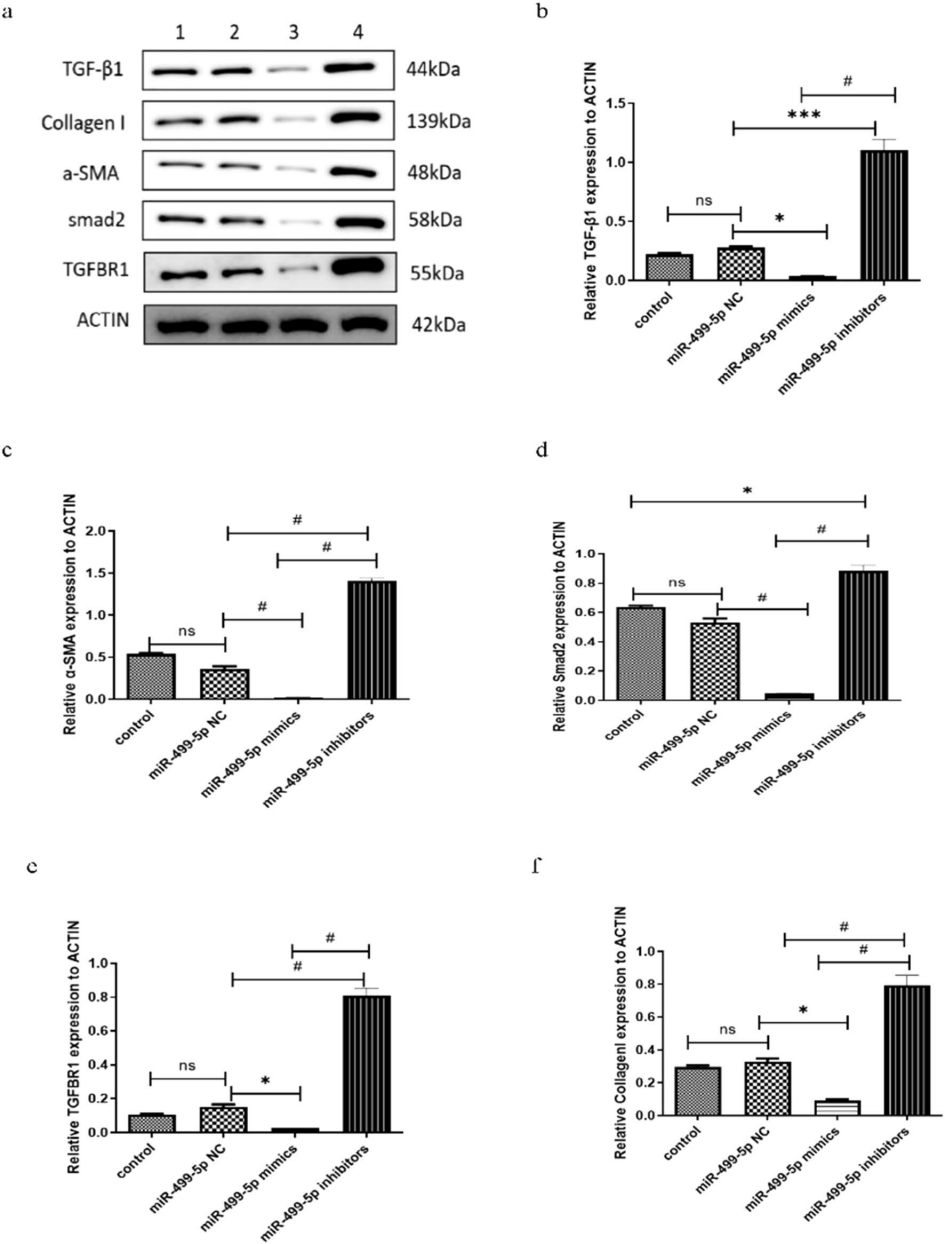
miR-499-5p inhibited the TGF-β/Smads signaling pathway and collagen secretion in protein level. **a** Expression levels of TGF-β1, TGFB-β1, smad2, α-SMA and collagen-I proteins in atrial fibro-blasts. 1, Control group; 2, miR-499-5p NC group; 3, miR-499-5p minic group; 4, miR-499-5p inhibitor group. **b** Relative expression of TGF-β1 level by qRT-PCR. *P < 0.05,***P < 0.001, ^#^P < 0.0001, ns: P > 0.05. **c** Relative expression of TGFB-β1 level by qRT-PCR. *P < 0.05, ^#^P < 0.0001, ns: P > 0.05. **d** Relative expression of Smad2 level by qRT-PCR. *P < 0.05, ^#^P < 0.0001, n: P > 0.05. **e** Relative expression of α-SMA level by qRI-PCR. *P < 0.05, ^#^P < 0.0001, ns: P > 0.05. **f** Relative expression of collagen-I level by qRT-PCR.*P < 0.05, ^#^P < 0.0001, ns: P > 0.05

The original article has been corrected.

